# Congenital perianal lipoma: a case report and review of the literature

**DOI:** 10.1186/s40792-019-0753-z

**Published:** 2019-12-16

**Authors:** Yudai Goto, Kazuaki Takiguchi, Hirofumi Shimizu, Hayato Go, Hideaki Tanaka

**Affiliations:** 1 Department of Pediatric Surgery, Fukushima Medical University Hospital, 1 Hikarigaoka, Fukushima, Fukushima, 960-1295 Japan; 2 Department of Pediatrics, Fukushima Medical University Hospital, 1 Hikarigaoka, Fukushima, Fukushima, 960-1295 Japan

**Keywords:** Neonate, Lipoma, Perineal, Perianal, Prenatal ultrasound

## Abstract

**Background:**

The surgical strategy for congenital perineal lipoma varies depending on the size, location, and accompanying congenital anomalies, with the optimum approach remaining to be determined. We herein report a case of congenital perianal lipoma that was first detected by prenatal ultrasound and review the literature.

**Case presentation:**

A female neonate was referred to us for the evaluation of a perianal mass. She had been considered to be male prenatally because fetal ultrasound showed a perineal mass similar to a scrotum and penis. A postnatal examination revealed an appropriate-for-age neonate with a soft round mass 1.5 cm in diameter just to the left of the anal verge. She passed urine and stool smoothly, and contrast enema confirmed no anorectal malformation. Magnetic resonance imaging showed that the lesion had a signal intensity consistent with fat located close to the anal sphincter, and no spinal anomaly (e.g., spina bifida) was identified. We excised the lesion (pathologically confirmed to be lipoma) simply at 2 months old, taking care to avoid damaging the anal sphincter by using a muscle stimulator. She has been doing well with good bowel movement and satisfactory cosmetic results for a follow-up period of one and a half years.

Our literature search revealed 49 cases of perineal lipoma reported in English in the last 25 years, and 74% of them—including ours—had other congenital anomalies, the breakdown of which was anorectal malformation in 40% of cases, labioscrotal fold or accessory scrotum in 28%, and urogenital malformation, congenital pulmonary airway malformation, and disorder of sex differentiation. The prenatal detection of the lesion, as in our case, was quite rare.

**Conclusion:**

A thorough physical examination after birth, magnetic resonance imaging and contrast enema to identify the nature of the perineal lipoma and accompanying anomalies are crucial for planning the surgical strategy. The lesion may be deeply interspersed between the sphincter muscle, especially when it accompanies anorectal anomaly. A muscle stimulator is useful for preserving and repairing the sphincter muscles during resection in order to ensure satisfactory bowel movement.

## Background

Congenital perineal lipoma, including perianal lesions, has been reported in about 50 cases in English literatures [1–26]. Its size and location vary among patients, and it sometimes accompanies other anorectal and/or urogenital anomalies. The surgical strategy for congenital perineal lipoma may therefore differ depending on such factors, with the optimum approach remaining to be determined.

We herein report a case of congenital perianal lipoma that was first detected by prenatal ultrasound. The prenatal detection or diagnosis of such lesions is quite rare [8, 14, 23, 25]. We will discuss the appropriate surgical strategy for such lesions and review the literature.

## Case presentation

**Fig. 1:**
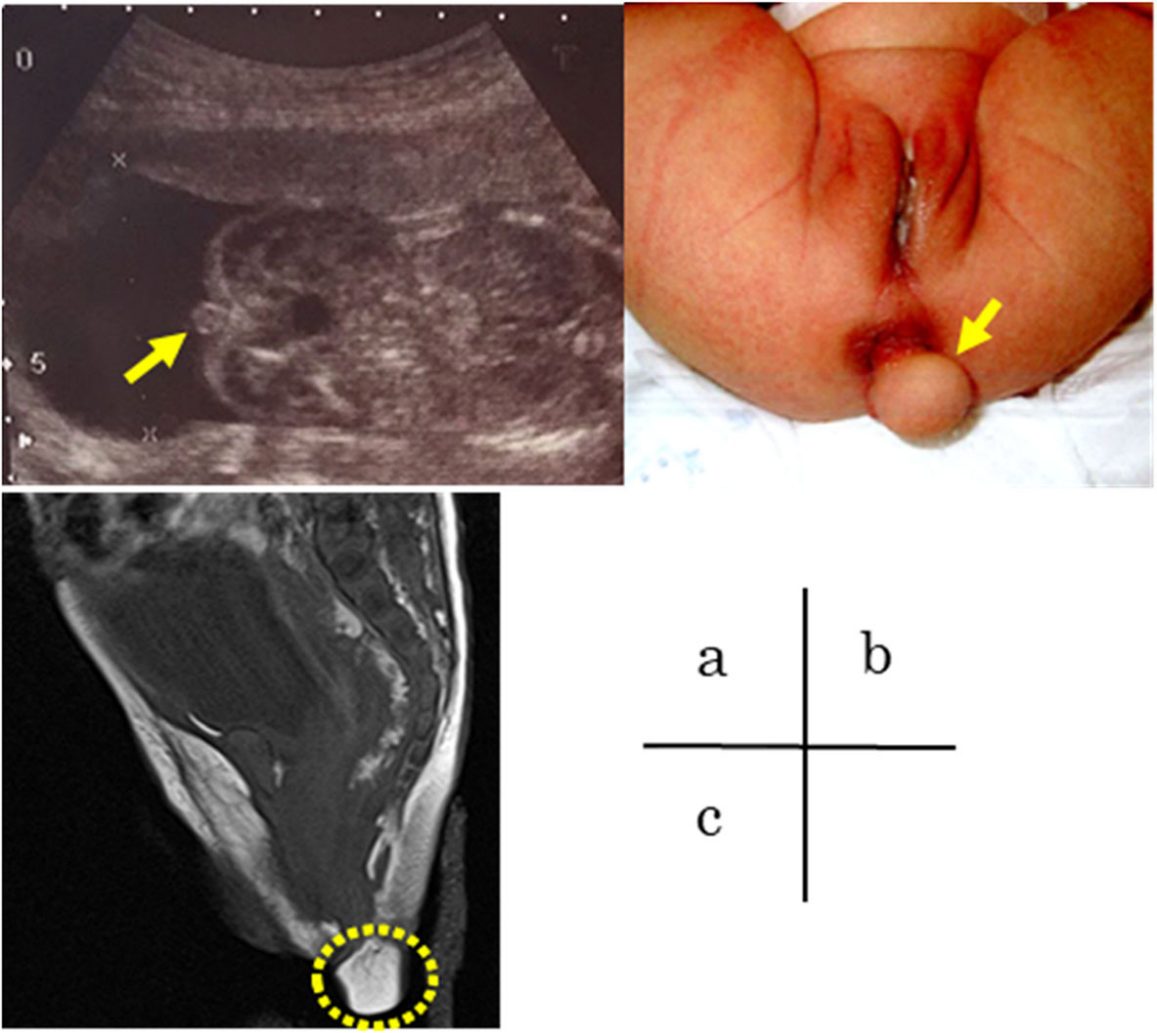
**a** Prenatal ultrasound showing the perineal lipoma (arrow) at gestational age of 20 weeks and 4 days. **b** Postnatal appearance of the soft round mass just left of the anal verge (arrow). **c** T2-weighted magnetic resonance imaging (sagittal view) of the perianal mass (dotted circle) in indicating a signal intensity consistent with fat located close to anus, and no spinal anomaly

A female neonate was referred for the evaluation of a perianal mass. She was delivered at 39 weeks and 1 day of gestation weighing 3097 g. The Apgar score was 9 at both 1 and 5 min after birth. She had been considered male prenatally because fetal ultrasound showed a perineal mass similar to male genitalia at a gestational age of 20 weeks and 4 days (Fig. 1a). A postnatal examination revealed an appropriate-for-age neonate with a soft round mass 1.5 cm in diameter just left of the anal verge (Fig. 1b). She passed urine and stool smoothly. Complete blood counts, electrolytes, liver and renal function tests, and urinalysis findings were all within normal ranges. Contrast enema confirmed no anorectal malformation (ARM). Magnetic resonance imaging (MRI) showed that the lesion had a signal intensity consistent with fat located close to the anal sphincter, and no spinal anomaly (e.g., spina bifida) was identified (Fig. 1c). The operative procedures at 2 months of age were as follows: the skin incision was made around the bottom of the pedicle of the lesion. The margin between the tumor and the surrounding subcutaneous fat tissue was not clear, but the tumor did not invade into the sphincter muscle. The lesion was resected with some of the subcutaneous tissue attached, and with small area of the surface of external sphincter muscle exposed. An electrical muscle stimulator was used to confirm that the muscle was left intact. The skin was closed with interrupted sutures.

A histologic examination of the specimen showed mature adipose tissue interspersed with collagenous bands, leading to a diagnosis of lipoma. She was discharged from the hospital without any complication and has been doing well with good bowel movement and satisfactory cosmetic results for a follow-up period of one and a half years.

## Discussion

**Fig. 2:**
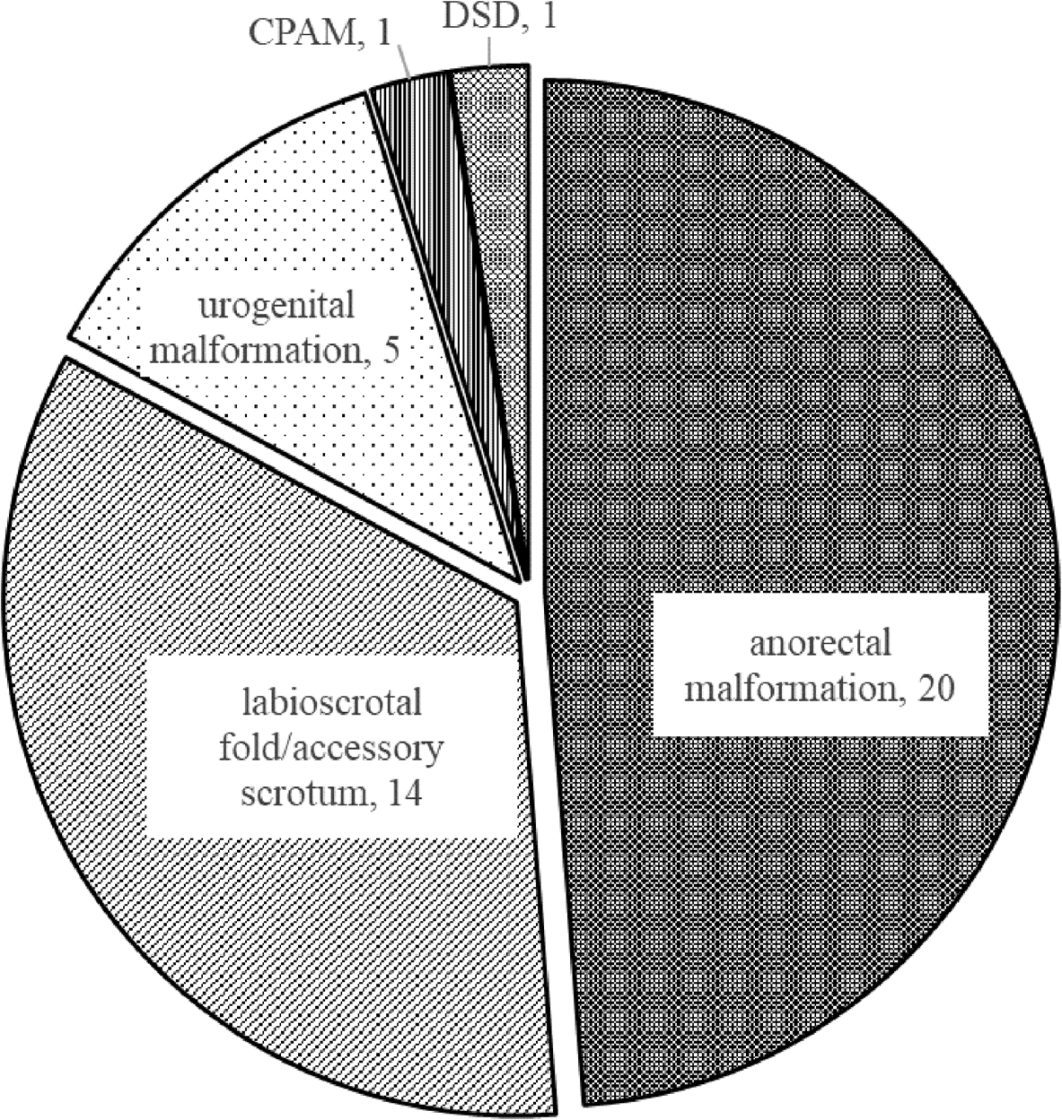
Breakdown of the congenital anomalies accompanied by perineal lipoma reported in the literatures. DSD, disorders of sex development; CPAM, congenital pulmonary airway malformation

We conducted a literature search using PubMed with keywords of lipoma, perianal, perineal, and neonate, identifying 49 cases of this lesion reported in English since 1994 [1–25]. The clinical features of the 50 total cases, including ours, are described in Table 1. There were 25 males and 25 females, and 37 cases (74%) accompanied other anomalies (breakdown shown in Fig. 2), including ARM most frequently (40%), followed by labioscrotal fold or accessory scrotum (28%), and urogenital malformation, congenital pulmonary airway malformation, and disorder of sex differentiation. The perianal lesion in our case was detected by a referring obstetrician using fetal ultrasound but was initially considered to be male genitalia; however, a correct diagnosis could have been achieved with more thorough assessments. Our literature search revealed only four cases in which perineal lesions were detected prenatally: case no. 34 and no. 45 in Table 1 were diagnosed as lipoma by fetal ultrasound and MRI, while case no. 35 was diagnosed with ultrasound only, and case no. 27 was detected as an uncharacterized mass using fetal ultrasound and MRI at gestational ages of 31, 32, 31, and 25 weeks, respectively. Two of these cases were found to have hypospadias and/or accessory scrotum after birth.

**Table 1:** Characteristics of the patients with perineal lipoma reported in English literature from 1994 to 2019. *DSD*, disorders of sex development; *CPAM*, congenital pulmonary airway malformation

Case	Author	Year	Sex	Size (cm×cm)	Age	Anomaly	Prenatal finding
1	Sule	1994	f	3.5×2	1m	none	none
2	Sule	1994	m	2.5×2	3w	accessory scrotum	none
3	Chanda	2000	f	4×2	unknown	none	none
4	Redman	2001	f	3×3×3	unknown	accessory labioscrotal fold	none
5	Ogasawara	2001	f	4	3m	none	none
6	Shaul	2005	m	unknown	unknown	rectourethral fistula	none
7	Shaul	2005	m	unknown	unknown	rectourethral fistula	none
8	Shaul	2005	m	unknown	unknown	rectourethral fistula	none
9	Shaul	2005	m	unknown	unknown	imperforate anus	none
10	Shaul	2005	f	unknown	unknown	cloaca	none
11	Shaul	2005	f	unknown	unknown	cloaca	none
12	Shaul	2005	f	unknown	unknown	cloaca	none
13	Shaul	2005	f	unknown	unknown	cloaca	none
14	Shaul	2005	f	unknown	unknown	rectovestibular fistula	none
15	Shaul	2005	f	unknown	unknown	rectovestibular fistula	none
16	Park	2006	m	unknown	2y	undescended testis, hypospadias, accessory scrotum	none
17	Park	2006	m	3×4	7m	accessory scrotum	none
18	Park	2006	m	7×3	4m	accessory scrotum	none
19	Park	2006	m	unknown	1y	none	none
20	Park	2006	m	unknown	9m	rectourethral fistula, hypospadias	none
21	Wester	2006	m	unknown	1y	rectourethral fistula	none
22	Wester	2006	m	unknown	4m	rectourethral fistula	none
23	Wester	2006	m	unknown	1y	rectovesical fistula	none
24	Wester	2006	f	unknown	5y	cloaca	none
25	Wester	2006	f	unknown	3m	rectovestibular fistula	none
26	Wester	2006	f	unknown	1y	rectovestibular fistula	none
27	Bataille	2007	m	2	unknown	none	para-anal nodular pediculated tumor
28	Guerra-Junior	2008	f	3×2×1.5	17d17d	none	none
29	Guerra-Junior	2008	f	1.5×1.5×1	2m	imperforate anus	none
30	Mohta	2008	m	3 to 4	5m	none	none
31	Harada	2009	m	unknown	4y	accesory scrotum	none
32	Soccorso	2009	m	4×4	unknown	accessory scrotum	none
33	Chu	2009	f	3×2	unknown	accessory labioscrotal fold, anovestibular fistula	none
34	Nakamura	2010	m	3.6×1.4×3.5	-	accessory scrotum, hypospadias	para-anal echogenic mass, suspected of lipoma
35	Wax	2010	f	5×4×3	1m	none	rt. labial and perineal avascular mass, suspected of lipoma
36	Numajiri	2011	f	2×1.1	4y	accessory labioscrotal fold	none
37	Numajiri	2011	f	3.2×1.9	7y	none	none
38	Numajiri	2011	f	3×4.5	3y	none	none
39	Kavecan	2012	m	3	1m	accessory scrotum	none
40	Chatterjee	2012	m	unknown	1y	accesory scrotum	none
41	Mahalik	2013	f	unknown	unknown	DSD, anterior ectopic anus	none
42	Periquito	2014	m	1.38	6m	none	none
43	Mifsud	2014	f	5×2.8	1y6m	accessory labioscrotal fold, unilateral renal agenesis	none
44	Iida	2014	m	3×3×2	1y6m	none	none
45	Murase	2015	m	2	1m	accessory scrotum	mass posterior to the scrotum, suspected of lipoma
46	Kim	2016	m	5×1	7m	none	none
47	Futhaddin	2018	m	1.5×1.5	6m	accesory scrotum, CPAM	none
48	Hashizume	2018	f	1	6m	accessory labioscrotal foldanovestibular fistula	none
49	Hashizume	2018	f	3	23d	anovestibular fistula	none
50	Presented case	2019	f	1.5×1.5	2m	none	considered as scrotum

The differential diagnoses of perineal lesions other than lipoma include lipoblastoma, sacrococcygeal teratoma, infantile hemangioma, hamartoma, choristoma, liposarcoma, enterogenous cyst, and ambiguous genitalia [8, 15]. If fetal ultrasound reveals abnormal genitalia, fetal MRI is needed in order to clearly define the lesion and other potential anomalies. Prenatal diagnosis or detection of a mass or abnormality at the genitalia should have a major impact on perinatal surgical or medical consult together with postnatal outcome. For example, pediatric surgeons need to evaluate ARM promptly to decide whether the newborn should have anoplasty soon after birth or start periodical bougie for low-type fistula, or have colostomy for male rectourethral fistula, soon after birth with delayed corrective surgery some months later. Congenital adrenal hyperplasia is one of the diseases that cause ambiguous genitalia and requires steroid-replacement therapy to prevent salt-wasting crisis. Regarding the delivery mode, one of the newborns whose perineal lesions were detected prenatally was delivered by cesarean section (one of twins, case no. 27), but the reason of the mode was not described in the paper. The maximum diameter of the reported cases was 5 cm (case no. 35, no. 43, no. 46), whose mode of delivery was vaginally or not described. If the prenatal imaging studies reveal that the diameter of the lesion is beyond the fetal head, or if the shape and vascularity of the lesion are highly indicative of injury of the lesion at the time of vaginal delivery, cesarean section would be more appropriate than vaginal delivery.

Postnatally, a careful physical examination for its precise location, size, and accompanying anomalies is crucial to achieving a correct diagnosis. These lesions are typically lobulated, round, or pedunculated subcutaneous masses that are smooth, soft, mobile, and nontender [8]. Postnatal urinary and meconium passage should be carefully observed in case the lesion obstructs those areas and requires prompt surgical intervention. Ultrasonography and MRI can help identify the internal fatty content and its anatomical relationship with the surrounding structures, along with potential complications [8, 23]. Contrast enema is also useful for ruling out anorectal anomalies and/or bowel obstruction, especially when the lesion is close to the anus, as in our case. Contrast-enhanced computed tomography may be needed when the above-mentioned imaging studies are insufficient to diagnose the lesion and/or other anomalies. If a neonate has no other anomalies other than the perineal lipoma as in our case, the timing of the surgery would be decided depending on two factors: the mass-effect causing urinary and/or intestinal obstruction, and technical difficulty in early surgery. The former would require surgery soon after birth. Regarding the latter, if the lesion is located close to vagina, for example, it would be appropriate for the operation to be delayed for about 3 months or later, which would make the precise dissection between the lesion and the vagina easier than doing in early neonatal period.

On the other hand, most reported lesions accompanying anorectal anomalies have been resected at the time of anorectal reconstruction [5, 14]. One female with anovestibular fistula had a lipoma (3 cm in diameter) close to the fistula; it was partially resected during the neonatal period to facilitate the passage of stool, followed by complete resection at the time of corrective surgery for the ARM [26]. Dissection of the perianal lesion from subcutaneous tissue, especially sphincter muscles, was successfully performed with the aid of a muscle stimulator in our case. Some perineal lipomas complicated by ARM are reportedly interspersed between the sphincter muscles [5] and may have a negative impact on the bowel functional outcome [7]. Special care should be taken to preserve the external anal sphincter using an electrical muscle stimulator during resection [5], and incised muscles or structures should be repaired accordingly.

## Conclusion

We herein report a rare case with prenatally detected perianal lipoma that neonatologists and pediatricians should note. Surgical consultation should be sought, and thorough perinatal investigations of the lesion and associated anomalies are crucial for planning surgery. An electrical muscle stimulator is useful for preserving the anal sphincter during resection to achieve satisfactory bowel movement.

## Data Availability

We would not like to share data other than those described in the paper, because they include personal information.

## Abbreviations

ARM: Anorectal malformation
MRI: Magnetic resonance imaging
DSD: Disorders of sex development
CPAM: Congenital pulmonary airway malformation
